# Geometric Accuracy Improvement by Using Electrochemical Reaming with a Helical Tube Electrode as Post-Processing for EDM

**DOI:** 10.3390/ma12213564

**Published:** 2019-10-30

**Authors:** Yan Zhang, Chen Wang, Yu Wang, Qin Ni, Lei Ji

**Affiliations:** 1 School of Mechanical and Power Engineering, Nanjing Tech University, Nanjing 211800, China; wangchen_fly@njtech.edu.cn (C.W.); niqinpanda@163.com (Q.N.); jilei.1993@163.com (L.J.); 2 AECC Sichuan Gas Turbine Research Establishment, Sichuan 610500, China; wangy_cgte@163.com

**Keywords:** EDM, electrochemical reaming, helical tube electrode, lateral flushing

## Abstract

Electrochemical reaming using a helical tube electrode together with lateral flushing is proposed as post-processing to improve the machining accuracy and surface quality of holes produced by electrical discharge machining (EDM). The velocity distributions of flushing in the machining gap for a cylindrical tube electrode and for a helical tube electrode were compared using flow field simulations. The role of the helical structure in promoting removal of machining products was illustrated by the results of the flow field simulations for different lateral flushing pressures. The performance of electrochemical reaming as post-processing in improving machining accuracy and surface quality was verified by comparative experiments examining the exit circularity error, taper, and surface morphology of machined holes. Finally, an optimum combination of machining parameters was obtained through a process parameter optimization experiment.

## 1. Introduction

Cobalt-based superalloys are widely used in engine turbine blades. To ensure the normal operation of such blades in a high-temperature environment, advanced film cooling technology [1] is applied. Film cooling holes are machined on the blade surface to reduce the surface temperature [2] and extend the working life of the blade [3]. At present, high-speed electrical discharge machining (EDM) is widely used for drilling film cooling holes because of its unique advantages, such as high machining speed [4] and accuracy [5] and the absence of residual stresses [6]. However, owing to the lack of working fluid in the lateral gap, especially when the hole is penetrated, there is not sufficient machining time for the exit, which leads to poor accuracy of the exit and a larger hole taper. Additionally, owing to the large amount of heat released in EDM, the hole wall is always covered with many surface defects, such as recast layers [7], cracks [8], and burrs [9], which are not acceptable in aerospace manufacturing. Therefore, EDM cannot be used to obtain film cooling holes with the required quality.

To improve machining accuracy and reduce hole surface defects, electrochemical machining (ECM) has been proposed as an alternative to EDM. During ECM, material is removed in the form of metal ions by electrochemical dissolution reactions, allowing a high surface quality to be achieved. Nguyen et al. [10] combined micro-EDM and micro-ECM in a unique hybrid machining process using low-resistivity deionized water and short voltage pulses. The application of ECM led to a reduction in surface roughness. Kurita and Hattori [11] reported the use of a complex machining technique combining EDM and ECM/ECM-lapping, in which the two processes were carried out in sequence with the same electrode and working liquid on the same machine tool. An EDM surface of 1 μm Ra was improved to 0.2 μm Ra by applying ECM. Wu et al. [12] proposed a method called wire electrochemical discharge machining (WECDM). Using this process, a rough machining surface with a recast layer caused by wire electrical discharge machining (WEDM) was removed by wire electrochemical machining (WECM). Han et al. [13] proposed a method using a powder-mixed electrolyte to improve the surface integrity of the electrochemical discharge machining (ECDM) process. The powder in the electrolyte stabilizes the discharge current through discharge energy dispersion, allowing better machining quality to be achieved. The inherent characteristics of ECM, namely, the absence of residual stress, tool wear [14], and surface defects [15], make it a feasible method for improving surface quality after EDM.

Hence, the combination of EDM and ECM is a promising method for the fabrication of microholes. However, in the combined EDM and ECM process, the removal of machining products from the narrow machining gap, which is key to good machining performance, poses a considerable challenge. Natsu et al. [16] utilized complex ultrasonic vibrations of the tool electrode to clean the machining gap in ECM by means of cavitation and stirring of the electrolyte. The accuracy of replication was improved by simultaneous longitudinal and lateral vibrations of amplitudes 10 mm and 4 mm, respectively. Qu et al. [17] reported the use of a reciprocating traveling wire electrode as the cathode in pulsed WECM of a slit. The homogeneity of the slit was improved through enhanced elimination of machining products.

However, the above-mentioned methods share the common limitation that it is difficult to drag products out from the machining gap when a tool electrode with a smooth surface is used. To overcome this problem, Liu et al. [18] investigated electrochemical drilling machining using a high-speed rotating helical electrode combined with an ultrashort voltage pulse, and reported the successful fabrication of a microhole structure with hole diameter of 186 μm and no taper. Wang et al. [19] showed experimentally that rotation of a helical electrode can promote the removal of electrolysis products from the machining gap, and that micro-through-holes with reduced overcut and taper could be obtained by ECM using a helical electrode. Hung et al. [20] developed a helical cylindrical electrode coated with a ceramic and epoxy double insulation layer for electrochemical microdrilling. The results obtained with this electrode indicated that the helical structure facilitated the removal of the processing products. Meanwhile, the insulation layer effectively avoided side machining. Fang et al. [21] proposed a method of wire electrochemical micromachining (WECMM) using a rotary helical electrode to enhance electrolyte refreshment in the machining gap. Both the maximum electrode feed rate and uniformity of slit width were effectively improved. Liu et al. [22] developed a method for processing microstructures on a glass material using WECDM with a solid helical tool. Their research focused on the impact of processing parameters on machining localization. A complex closed structure, a high-aspect-ratio kerf, and a patterned structure with high aspect ratio were fabricated successfully using optimized values of these parameters. Plaza et al. [23] proposed a new method based on the use of helical electrodes in micro-EDM drilling that was able to effectively remove the debris generated as the hole’s depth increased. The influences of helix angle and flute depth on process performance were addressed. Hung et al. [24] presented a novel technique utilizing micro-EDM combined with ultrasonic vibration of a helical microtool electrode to drill and finish microholes. They verified that microholes with good surface quality and reduced taper could be obtained through appropriate choices of parameters such as step variation, rotational speed, and ultrasonic amplitude.

In this paper, with the aim of further improving machining accuracy, especially for hole exits and machined surfaces, electrochemical reaming using a helical tube electrode together with lateral flushing is proposed as a post-processing method to finish holes machined by EDM. A series of investigations were conducted. First, the velocity distribution of flushing in the machining gap was analyzed by flow field simulation, with a comparison being made between a common tube electrode and a helical tube electrode. The function of the helical structure in promoting the removal of machining products was illustrated by flow field simulation at different lateral flushing pressures. Moreover, the performance of post-processing electrochemical machining in improving machining accuracy and surface quality was verified by a comparative experiment investigating the shape accuracy, dimensional accuracy, and surface morphology of holes. Finally, a combination of optimized machining parameters was obtained through a process parameter optimization experiment on electrochemical reaming with lateral flushing.

## 2. Machining Principle of Electrochemical Reaming Using a Helical Tube Electrode as Post-Processing for EDM

**Figure 1:**
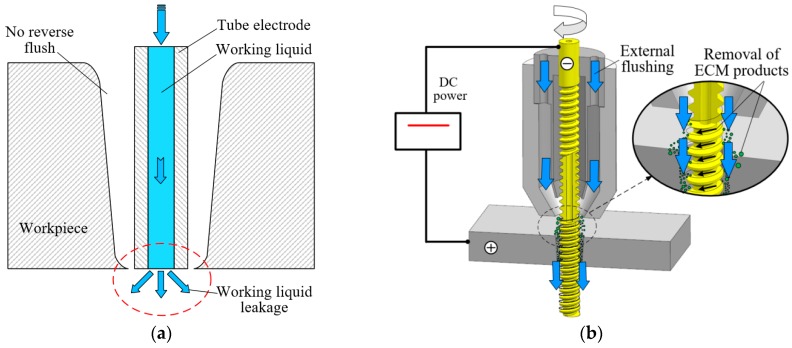
Schematic diagram of electrochemical reaming using a helical tube electrode together with lateral flushing: (**a**) after the electrical discharge machining (EDM) process; (**b**) electrochemical reaming as post-processing. ECM: electrochemical machining.

During the EDM process, because the tube electrode is used as the tool electrode, there is inevitably leakage of working fluid from the lateral gap at the moment at which the hole is pierced, as shown in Figure 1a. The diminishing amount of working fluid in the lateral machining gap will lead to insufficient machining and arc discharge. As a consequence, the accuracy of the exit shape will be severely reduced and the hole will have a greater taper, owing to the shorter time for processing the exit. To overcome these problems, electrochemical reaming using a helical tube electrode together with lateral flushing is proposed as a post-processing method, using the same machining station as for the EDM. Figure 1 shows a schematic diagram of electrochemical reaming using a helical tube electrode together with lateral flushing. After the hole has been pierced, the EDM process is completed, and the electrode stops feeding and comes to a halt. Then, with the application of DC power to the interelectrode gap and with flushing in the lateral gap, the ECM process is able to improve the machining accuracy, as shown in Figure 1b. The electrolyte (sodium nitrate solution) (Sinopharm Chemical Reagent Co., Ltd, Shanghai, China) flows into the machining gap from the entrance and flows out from the exit via the lateral gap. During this process, the helical tube electrode rotates counterclockwise, promoting electrolyte flow and removal of machining products, and thus enhancing the electrochemical reaction on the lateral walls of the hole.

## 3. Flow Field Simulation of Electrochemical Reaming with a Helical Tube Electrode

To illustrate the effect of a helical electrode in promoting the removal of machining products from the narrow machining gap, flow field simulations of ECM with lateral flushing using two different tool electrodes were carried out. Table 1 lists the parameters of the simulations.

**Figure 2:**
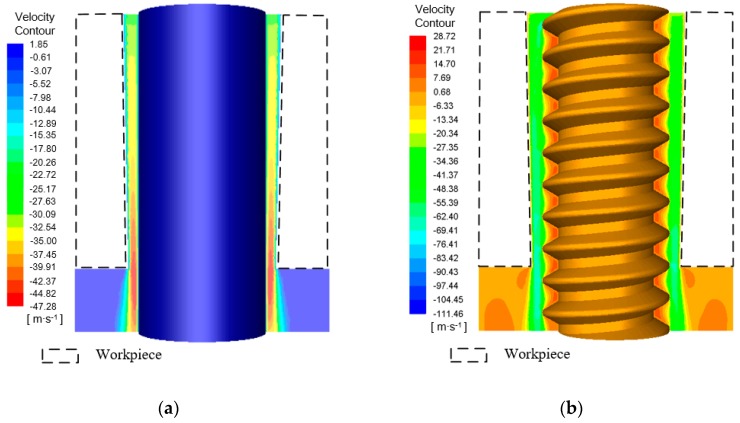
Velocity contours in the machining gap using different tube tool electrodes: (**a**) cylindrical tube electrode; (**b**) helical tube electrode.

Figure 2 shows the electrolyte axial velocity in the machining gap of ECM with lateral flushing using two different tool electrodes. It can be seen that there were obvious differences in the electrolyte velocity distribution in the machining gap with the different tube electrodes. When the cylindrical electrode was used, the velocity distribution in the machining gap near its exit was mostly concentrated at −39.91 m/s, as shown in Figure 2a. However, with the helical electrode, the electrolyte flow velocity outside the helical groove was mostly concentrated at −69.41 m/s, as shown in Figure 2b. In addition, it can be seen that the velocity distribution approached −14.7 m/s inside the helical groove. This indicates that for the same lateral flushing pressure and electrode rotation speed, the helical electrode led to a stronger disturbance of the flow field in the machining gap than the cylindrical electrode, and the resulting rapid flow velocity in the gap accelerated removal of electrolyte and machining products.

**Figure 3:**
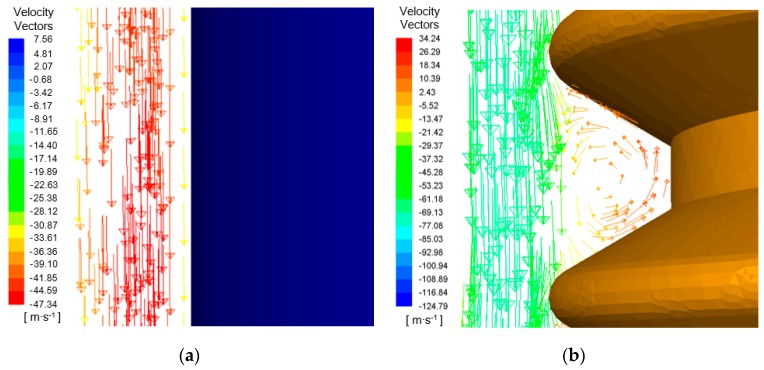
Velocity vectors in the axial direction in the machining gap using different tube tool electrodes: (**a**) cylindrical tube electrode; (**b**) helical tube electrode.

To further elucidate the effect of the helical structure on disturbing the flow field and accelerating the removal of machining products, the machining gap was further enlarged and the velocity vectors for the different electrodes were extracted, as shown in Figure 3. For the cylindrical tube electrode with a smooth surface, the velocity vectors in the machining gap pointed in the axial direction of the electrode, as shown in Figure 3a. However, for the helical tube electrode, although the velocity vectors in the machining gap outside the helical groove pointed in the direction of axial flushing, there were also velocity vectors representing counterclockwise rotation within the helical groove, as shown in Figure 3b. This indicates the presence of eddy flows in the helical groove, produced as a result of the strong disturbance of the electrolyte by the rotating helical structure. Thus, the machining products in the machining gap were entrained into the helical groove under the action of negative pressure, and they were then removed along the spiral direction by the downward axial force generated by the counterclockwise rotation of the electrode.

**Figure 4:**
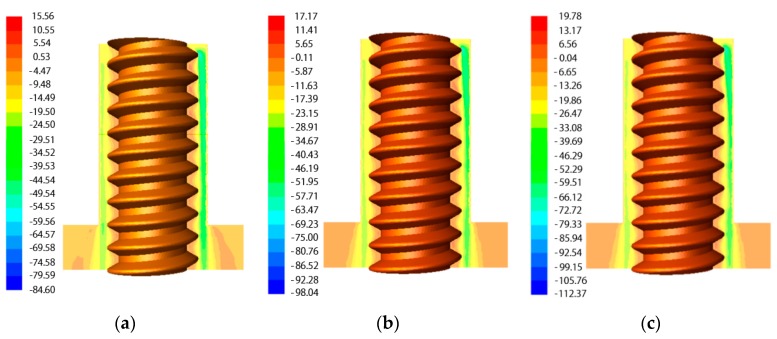

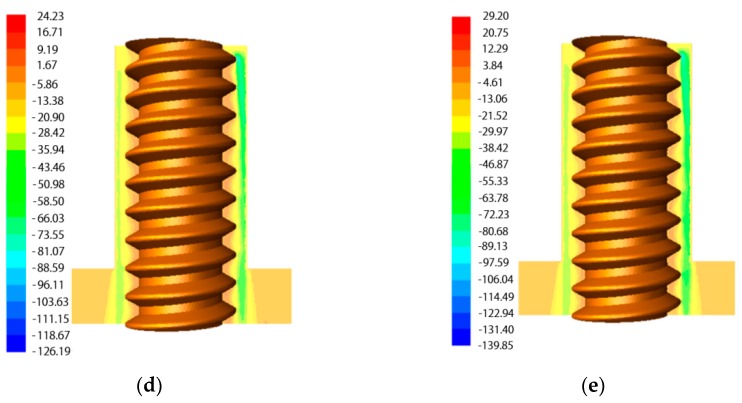
Contours of axial velocity in the machining gap during ECM at different lateral flushing pressures: (**a**) 1 MPa; (**b**) 2 MPa; (**c**) 3 MPa; (**d**) 4 MPa; (**e**) 5 MPa.

Figure 4 shows the contours of axial velocity in the machining gap at different lateral flushing pressures. It can be seen that the flushing pressure had an obvious influence on the velocity distribution in the machining gap during electrochemical reaming. With increasing flushing pressure from 1 MPa to 5 MPa, the electrolyte flow velocity increased significantly both inside and outside the helical groove. This implies that higher flushing pressures, in the range of 1–5 MPa, could further improve the flow of working fluid, thus effectively accelerating the removal of machining products.

**Figure 5:**
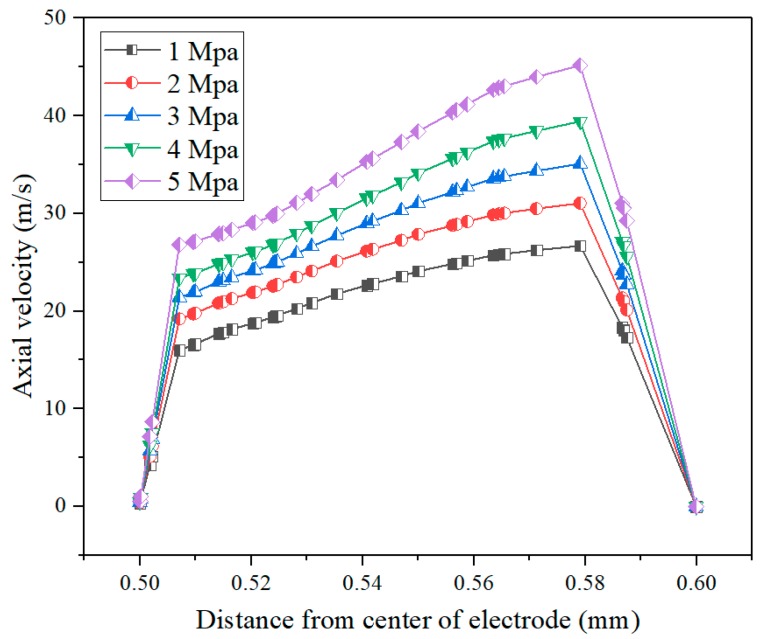
Axial velocity distribution of working fluid along sample lines at different flushing pressures.

Figure 5 illustrates the axial velocity distribution of the working fluid along sample lines at different flushing pressures during electrochemical reaming. With increasing lateral flushing pressure, the electrolyte flow velocity in the machining gap increased. Within the range of 1–5 MPa flushing pressure, the maximum flow velocity in the machining gap increased from 23 m/s to 45 m/s. Thus, increasing the flushing pressure was conducive to replenishing electrolyte in the machining gap. Within a distance from the center of electrode of 0.58–0.6 mm (in the helical grove), an increasing flow velocity was observed, which also indicated that an appropriate increase in the lateral flushing pressure can prevent the accumulation of machining products in the helical groove. Thus, the machining precision of electrochemical reaming was greatly improved through the enhanced flushing effect of the flow field.

## 4. Experimental Materials and Parameter Setup

**Figure 6:**
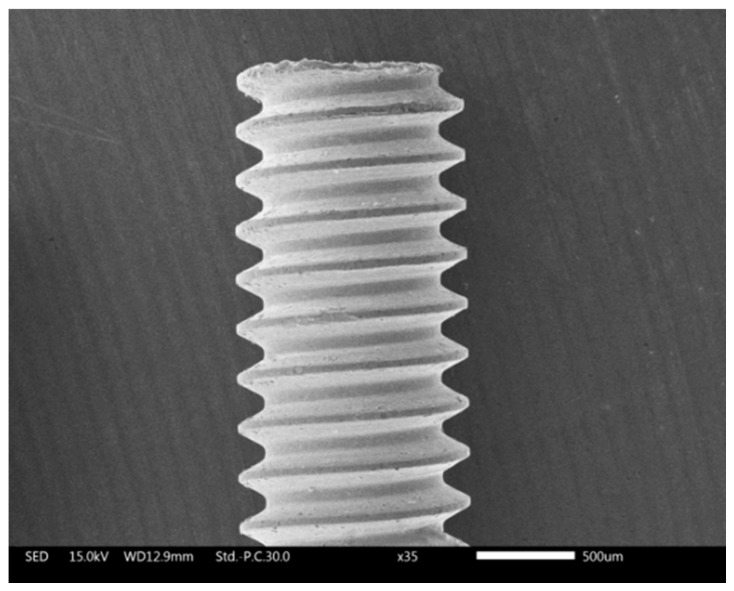
SEM (scanning electron microscope) image of the tube electrode with helical structure.

The tool electrode was a copper tube of 1 mm diameter with helical structure, as shown in Figure 6. A cobalt-based superalloy plate of thickness 2 mm was used as the workpiece. Deionized water (Shanghai Xunhui Enviroment Technology Co., Ltd, Shanghai, China) was used in the EDM process, and other processing parameters are listed in Table 2. The experimental parameters of the electrochemical reaming process are listed in Table 3.

**Figure 7:**
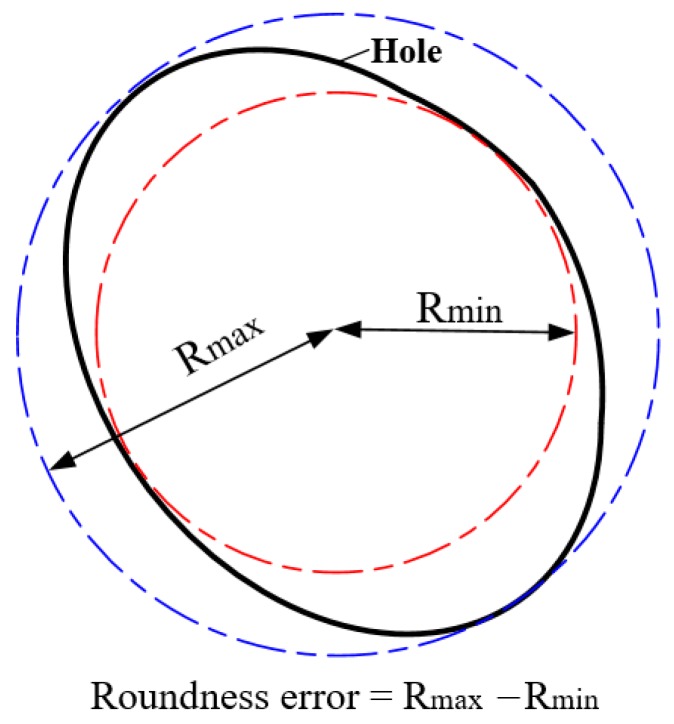
Roundness error calculation method.

The geometric parameters of the hole were measured using a scanning electron microscope (Japan Electronics Co., Ltd., Tokyo, Japan) and optical microscope (Leica, Hesse, Germany). The roundness error was calculated using the minimum region method, as shown in Figure 7.

The taper of the hole was calculated according to the following formula:

Tan*θ* = (*D*_entrance_ − *D*_exit_)/2*h*

where *D* is the diameter of entrance and exit and *h* is the thickness of workpiece.

## 5. Results and Discussion

### 5.1. Comparison of Shape Accuracy and Surface Quality of Holes Machined by Different Methods

**Figure 8:**
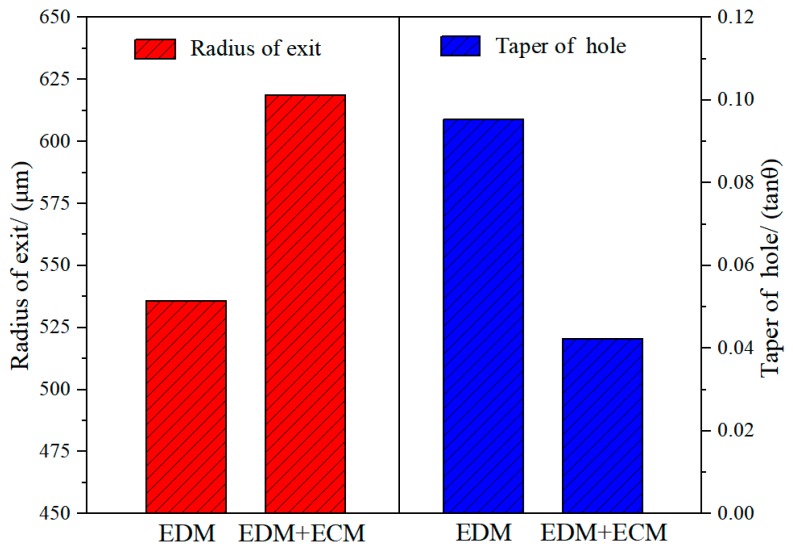
Geometric accuracy of holes machined by two different methods.

Figure 8 shows the geometric accuracy of holes machined by the two different methods, as represented by the exit diameter and the taper. With the use of electrochemical post-processing for EDM, the exit radius was increased from 538.86 μm to 615.84 μm, while the taper was reduced by more than 50%. This illustrates that the lateral flushing of the working fluid solved the problem of leakage of working fluid when the hole is pierced. Meanwhile, the use of a helical tube electrode accelerated the flushing of working fluid in the lateral gap. Thus, ECM is an effective method by which to further ream the exit and reduce the taper generated by EDM.

**Figure 9:**
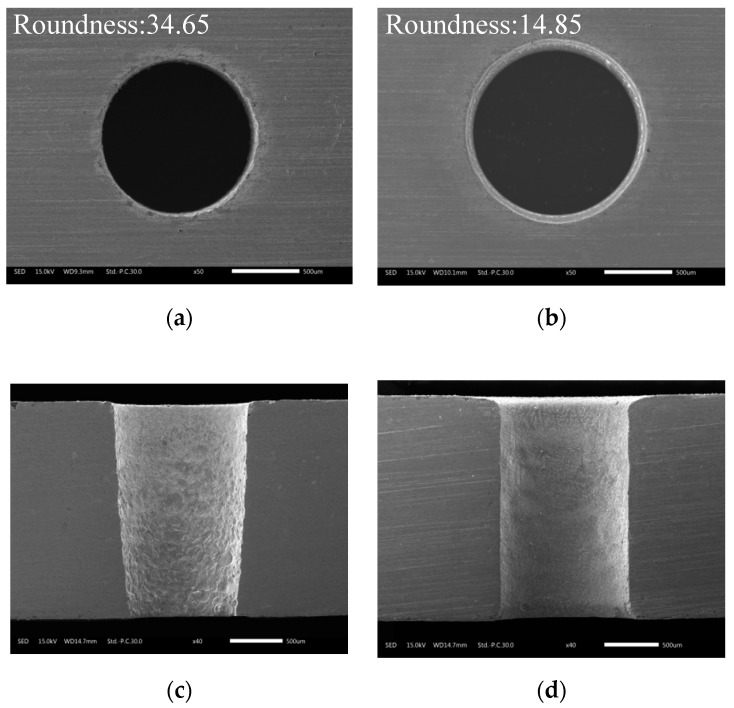
Morphology of (**a**,**b**) the exit and (**c**,**d**) the inner wall of holes machined by different methods: (**a**) without ECM; (**b**) after ECM post-processing; (**c**) without ECM; (**d**) after ECM post-processing.

Figure 9 shows the exit morphology, taper, and side-wall morphology of holes machined with and without ECM post-processing. By comparing the exit morphology of the holes in Figure 9a,b, it can be seen that the exit diameter of the hole machined by EDM was significantly enlarged after post-processing with electrochemical reaming. Moreover, the edge of the exit machined by EDM was rough and irregular, and the roundness was 34.65 μm, whereas after ECM post-processing, the edge was smooth and regular, and the roundness was reduced to 14.85 μm. As shown in Figure 9c, with EDM alone, owing to the lack of working liquid in the machining gap at the exit, a hole with a large taper angle was obtained. However, the taper angle was clearly improved after ECM, as shown in Figure 9d. These results, together with Figure 8, indicate that electrochemical post-processing with external flushing was able to further machine and improve the exit obtained by EDM, thereby enhancing the accuracy of the hole.

**Figure 10:**
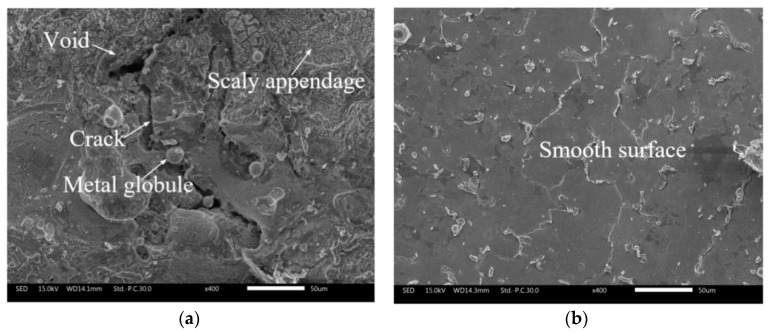
Comparison of cross-sectional surface morphology: (**a**) without ECM post-processing; (**b**) after ECM post-processing.

Figure 10 shows the surface morphology of the inner walls of holes machined by the different methods using a helical tube electrode. As can be seen from Figure 10a, the surface quality of the inner wall from EDM without ECM post-processing was extremely poor, with a large number of defects such as cracks, metal globules, and voids on the rough machined surface. In contrast, as shown in Figure 10b, the surface further processed by ECM with a helical tube electrode and external flushing was smooth, with a great reduction of the surface defects produced by EDM. There are two main reasons for the improvement in the surface quality of the hole wall. On the one hand, rapid lateral flushing of the electrolyte provided a clean and stable flow field for the whole electrochemical reaming stage, and continuous and sufficient electrochemical dissolution enabled effective removal of the surface defects caused by EDM. On the other hand, the rotating helical electrode greatly accelerated the removal of machining products, which further improved the rate of electrochemical reaction.

### 5.2. Improved Performance of ECM Post-Processing for EDM

#### 5.2.1. Effect of Applied Voltage of Electrochemical Reaming on Hole Accuracy

**Figure 11:**
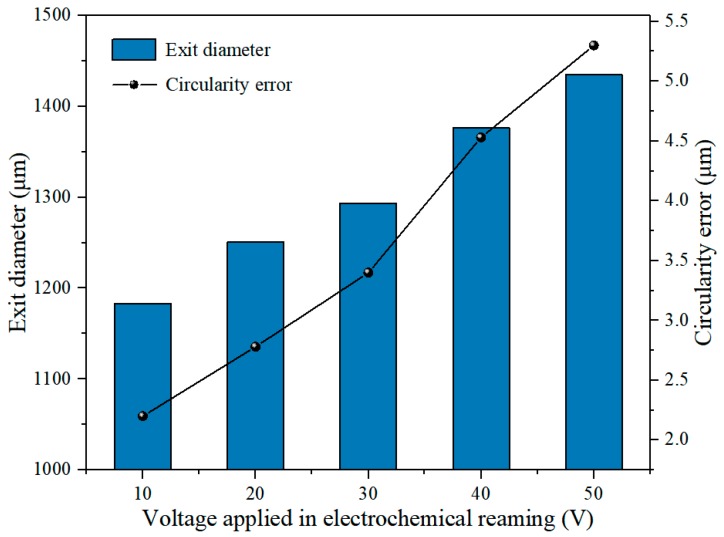
Variation of exit diameter and circularity error with applied voltage in electrochemical reaming.

**Figure 12:**
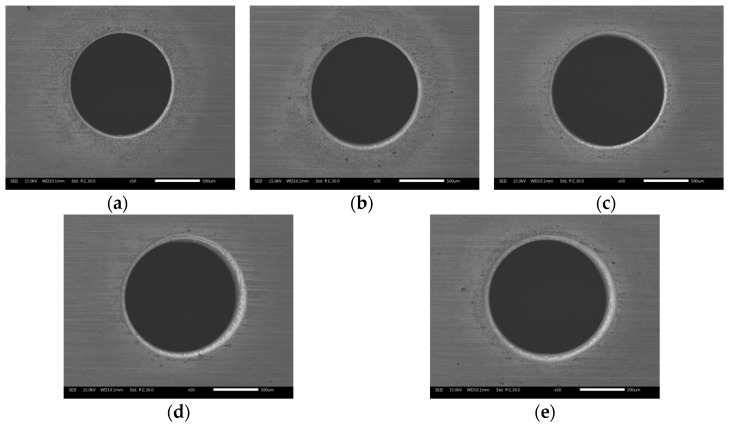
Morphology of the exit at different applied voltages in electrochemical reaming: (**a**) 10 V; (**b**) 20 V; (**c**) 30 V; (**d**) 40 V; (**e**) 50 V.

Figure 11 illustrates the effects of applied voltage on exit diameter and circularity error. It can be seen that both diameter and error increased linearly with increasing applied voltage. This is because the anodic dissolution rate increased with increasing voltage for the same electrolyte. The higher voltage increased the energy applied in the interelectrode gap, thus increasing the volume of material removed by electrochemical dissolution. The expansion rate of the side machining gap was accelerated owing to the increasing voltage, which eventually enlarged the diameter of the hole. The circularity error of the exit also increased with increasing applied voltage. In a sodium nitrate solution, a high voltage can lead to generation of numerous bubbles and machining products in the machining gap, possibly resulting in short circuit and sparking. Hence, the circularity of the exit can be destroyed by a high voltage. The morphology of the exit at different applied voltages, shown in Figure 12, also revealed that an excessive voltage was not conducive to uniform removal of material at the exit.

**Figure 13:**
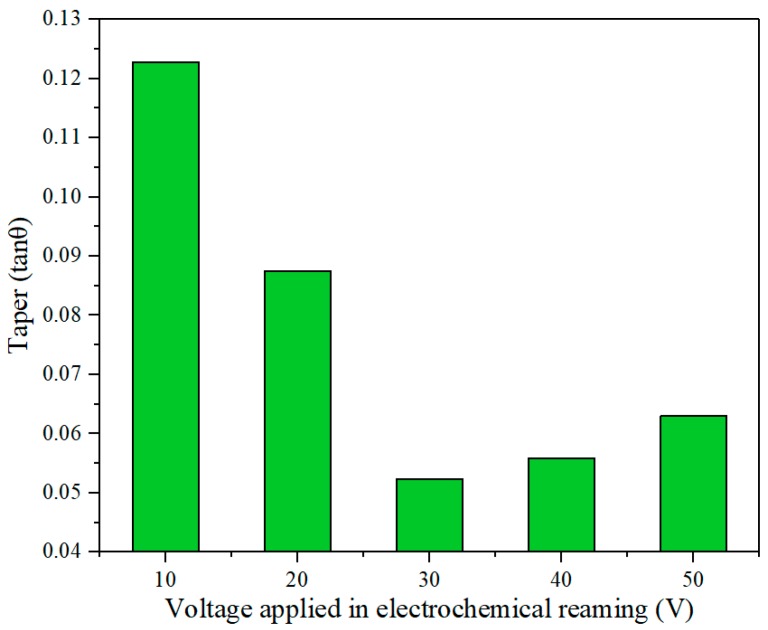
Effect of applied voltage on hole taper in electrochemical reaming.

**Figure 14:**
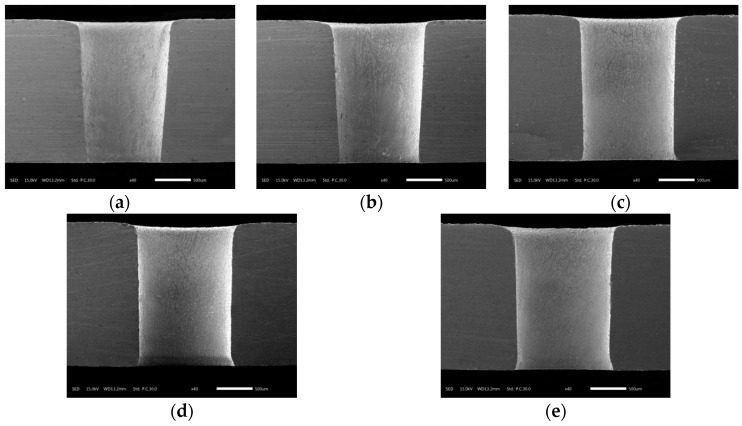
Sectional view of holes after electrochemical reaming at different applied voltages: (**a**) 10 V; (**b**) 20 V; (**c**) 30 V; (**d**) 40 V; (**e**) 50 V.

The hole taper gradually decreased as the voltage increased from 10 V to 30 V, but once the voltage exceeded 30 V, the taper tended to increase, as shown in Figure 13. The variation of the taper in Figure 13 was consistent with the sections of the holes shown in Figure 14. This is because when the machining gap at the exit was smaller than that at the entrance, the difference of machining gap between entrance and exit led to a difference of electric field intensity. Thus, the electric field intensity at the exit was larger under the same voltage. The machining gap at the exit was smaller and the electric field intensity was larger; thus, electrochemical dissolution occurred at the exit prior to the entrance. Hence, the taper decreased as the voltage increased between 10 V and 30 V. However, an excessive voltage (30–50 V) caused a large number of bubbles and hydroxide electrolytes to be generated in the helical groove and the machining gap. The deteriorating flow field directly resulted in unstable machining and increased hole taper.

The principal indices for evaluating the accuracy of the holes were taper, circularity error, and diameter. A hole with the minimum taper was obtained at 30 V applied voltage, according to Figure 13, and this voltage also gave acceptable circularity error and exit aperture, as shown in Figure 11. Hence, an applied voltage of 30 V was concluded to be optimal for the accuracy of the machined hole.

#### 5.2.2. Effect of Electrolyte Concentration in Electrochemical Reaming on Hole Accuracy

**Figure 15:**
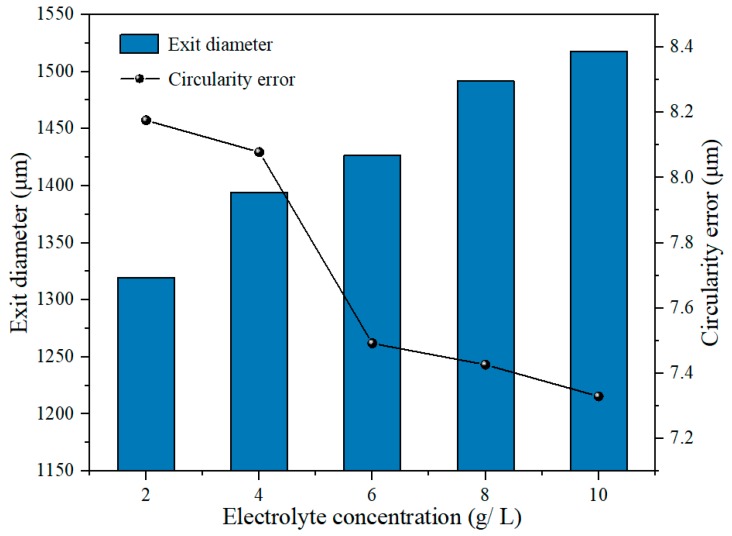
Variation of exit diameter and circularity error with electrolyte concentration in electrochemical reaming.

**Figure 16:**
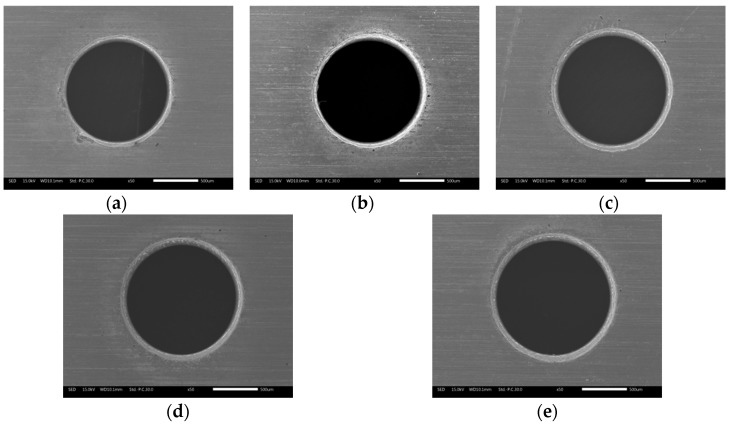
Exit morphology of holes after electrochemical reaming at different working fluid concentrations: (**a**) 2 g/L; (**b**) 4 g/L; (**c**) 6 g/L; (**d**) 8 g/L; (**e**) 10 g/L.

Figure 15 shows the variation of exit diameter and circularity error with electrolyte concentration in electrochemical reaming. It can be seen that the exit diameter increased with increasing electrolyte concentration, while the circularity error of the exit decreased. This is because the higher the electrolyte concentration, the higher is the conductivity of the solution. With other machining parameters remaining constant, the current density in the machining gap increased with increasing conductivity, which led to an increasing volume of material being removed by electrochemical reaction. The diameter of the exit was also gradually enlarged by enhanced electrochemical reaming. As this electrochemical expansion of the exit increased with further increase in concentration, the sharp edges and roundness faults generated by EDM were effectively removed. Thus, the machining accuracy as represented by the circularity of the exit was gradually improved as the concentration increased, as can be seen from the exit morphology in Figure 16.

**Figure 17:**
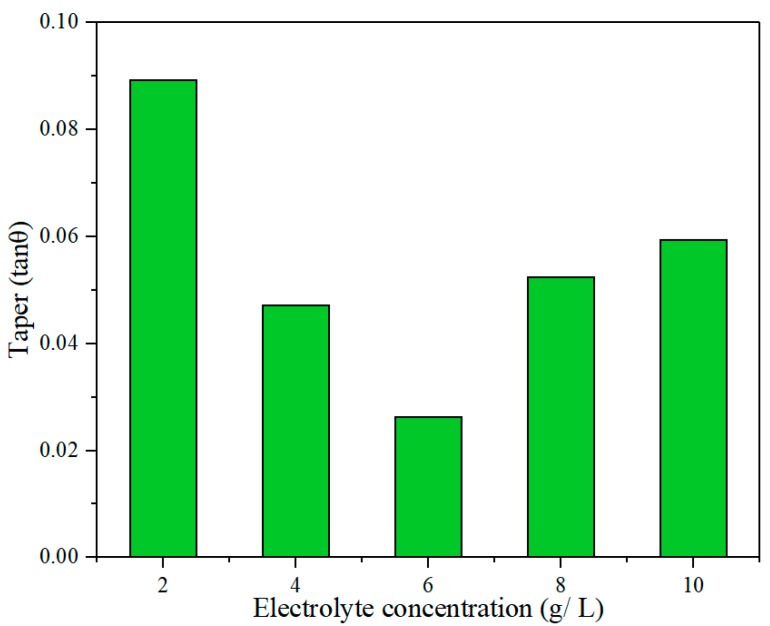
Effect of electrolyte concentration on hole taper in electrochemical reaming.

**Figure 18:**
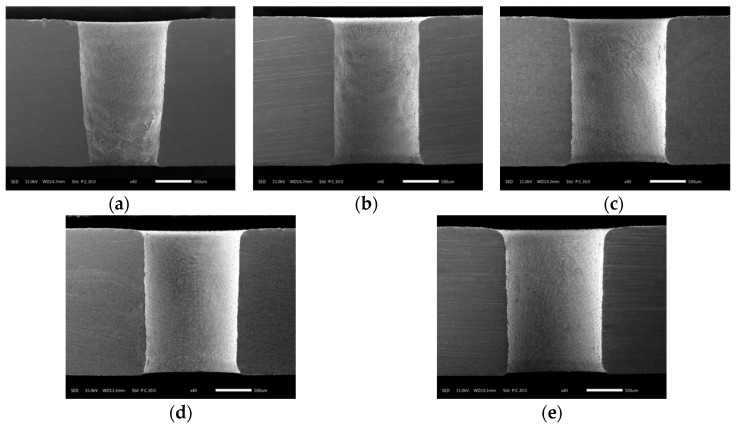
Sectional views of holes at different electrolyte concentrations in electrochemical reaming: (**a**) 2 g/L; (**b**) 4 g/L; (**c**) 6 g/L; (**d**) 8 g/L; (**e**) 10 g/L.

Figure 17 shows the variation of hole taper with electrolyte concentration. It can be seen that the taper first decreased and then increased with increasing concentration. Owing to the enhanced electrochemical reaction as the concentration increased, the exit was enlarged faster than the entrance, which reduced the taper. However, if the concentration was too high, products of electrochemical reaction accumulated at the exit, hindering uniform corrosion of the lateral wall of the hole and eventually resulting in an increased taper. This variation of the taper was consistent with the sectional views of the holes in Figure 18.

A hole with minimum taper and moderate circularity error and acceptable exit diameter was obtained at an electrolyte concentration of 6 g/L, which was therefore concluded to be the optimum electrolyte concentration for electrochemical post-processing.

#### 5.2.3. Effect of Duration of Electrochemical Reaming on Hole Accuracy

**Figure 19:**
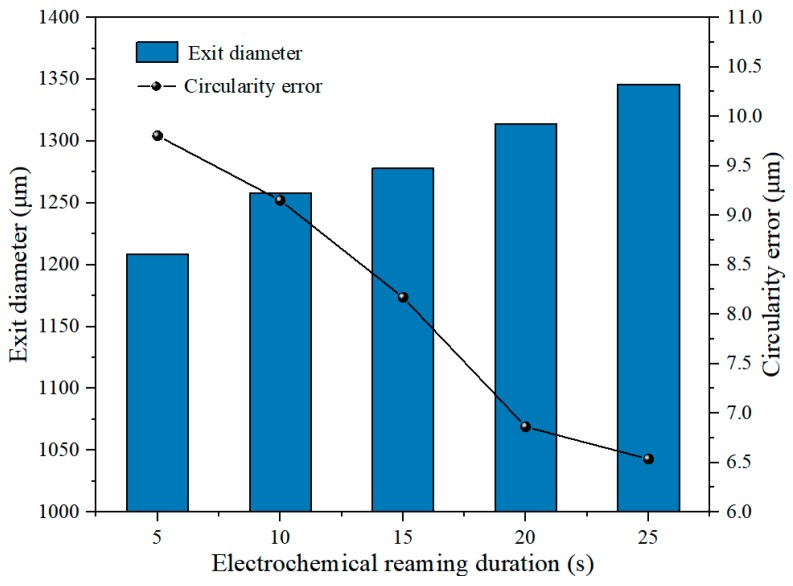
Effect of duration of electrochemical reaming on exit diameter and circularity error.

**Figure 20:**
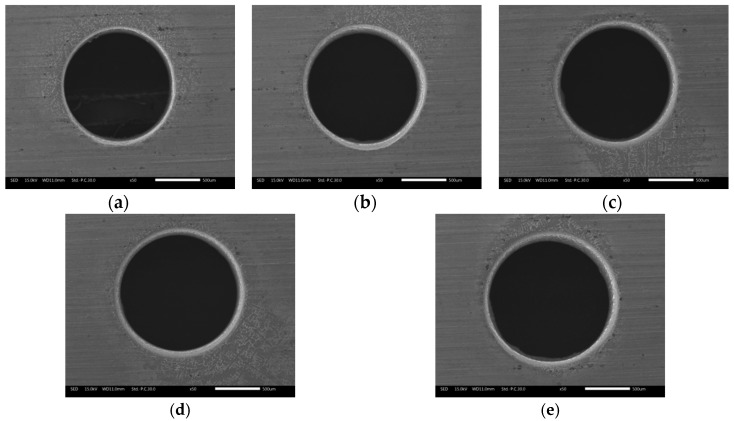
Exit morphology of holes after electrochemical reaming for different durations: (**a**) 5 s; (**b**) 10 s; (**c**) 15 s; (**d**) 20 s; (**e**) 25 s.

Figure 19 shows the variations of exit diameter and circularity error with the duration of electrochemical reaming. It can be seen that the exit diameter increased with increasing duration, while the circularity error of the exit gradually decreased. The increase in exit diameter is attributable mainly to the rapid enlargement of the lateral machining gap caused by electrochemical reaming. Simultaneously, the continuous electrochemical dissolution with increasing duration removed the defects at the exit machined by EDM and improved its circularity. Variations in the exit diameter and the circularity error with increasing duration of electrochemical reaming can also be identified in the exit morphologies shown in Figure 20.

**Figure 21:**
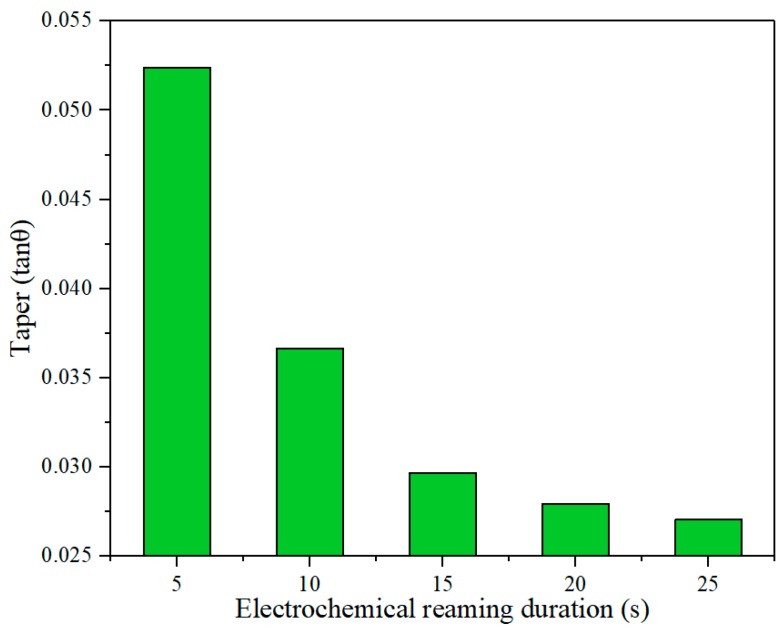
Effect of duration of electrochemical reaming on hole taper.

**Figure 22:**
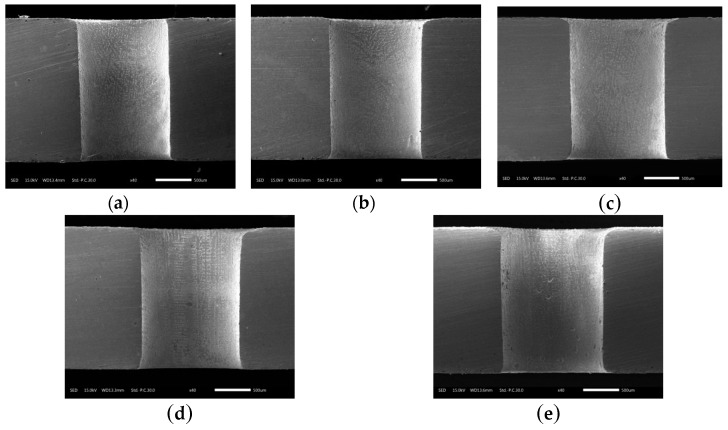
Sectional views of holes after electrochemical reaming for different durations: (**a**) 5 s; (**b**) 10 s; (**c**) 15 s; (**d**) 20 s; (**e**) 25 s.

The variation of hole taper with duration of electrochemical reaming is shown in Figure 21. It can be seen that the taper generally decreased with increasing duration. This decrease can be attributed to faster dissolution at the exit driven by a higher electric field intensity than at the entrance. Thus, the size of the exit was expanded to be close to that of the entrance, and the taper was thereby reduced. This variation in hole taper is visually reflected in the sectional views in Figure 22.

According to Figure 19 and Figure 22, a duration of electrochemical reaming of 25 s gave a hole with the minimum taper and optimum circularity of the exit.

#### 5.2.4. Optimized Machining Parameters

Therefore, taking account of the effects of applied voltage, electrolyte concentration, and duration of electrochemical reaming on the accuracy of the machined hole, the final optimized set of machining parameters for electrochemical post-processing with a helical tube electrode were determined to be an applied voltage of 30 V, an electrolyte concentration of 6 g/L, and a duration of electrochemical reaming of 25 s.

## 6. Conclusions

In this study, focusing on improving the accuracy of holes machined by EDM, electrochemical reaming using a helical tube electrode was proposed as post-processing for EDM. The main conclusions can be summarized as follows:

- Simulations of the flow field in the gap show that, compared with an ordinary circular tube electrode, a helical tube electrode was better at promoting renewal of electrolyte. When the helical electrode was used to process the holes, the concentrated velocity distribution within the machining gap increased from 39.91 m/s to 69.41 m/s. Within the range of 1–5 MPa flushing pressure, the maximum flow velocity in the machining gap increased from 23 m/s to 45 m/s.
- Comparative experimental results show that post-processing with electrochemical reaming clearly improved the shape precision of holes, including the taper and exit circularity. With the use of electrochemical post-processing for EDM, the exit radius was increased from 538.86 µm to 615.84 µm, while the taper was reduced by more than 50%. Moreover, the surface defects in holes machined by EDM were effectively removed by electrochemical dissolution reactions.
- The optimum combination of machining parameters was found to be an applied voltage of 30 V, an electrolyte concentration of 6 g/L, and a duration of electrochemical reaming of 25 s.

## Figures and Tables

**Table 1:** Parameters of flow field simulation for different tool electrode conditions.

Parameter	Value
Electrode diameter	1 mm
Rotation speed of electrode	480 rev/min
Pressure of working fluid	3 MPa
Workpiece thickness	2 mm

**Table 2:** Experimental parameters of the electrical discharge machining (EDM) process.

Parameter	Value
Flushing pressure	4 MPa
Rotational speed of tool electrode	300 rev/min
Pulse duration	12 μs
Pulse interval	12 μs
Peak current	14 A

**Table 3:** Experimental parameters of the electrochemical reaming process.

Parameter	Value
Flushing pressure	4 MPa
Rotational speed of tool electrode	300 rev/min
NaNO_3_ electrolyte concentration	2, 4, 6, 8, or 10 g/L
Voltage amplitude	10, 20, 30, 40, or 50 V
Electrochemical reaming duration	5, 10, 15, 20, or 25 s
